# Interpretable Disorder Signatures: Probing Neural Latent Spaces for Schizophrenia, Alzheimer’s, and Autism Stratification

**DOI:** 10.3390/brainsci15090954

**Published:** 2025-09-01

**Authors:** Zafar Iqbal, Md. Mahfuzur Rahman, Qasim Zia, Pavel Popov, Zening Fu, Vince D. Calhoun, Sergey Plis

**Affiliations:** 1 Department of Computer Science, Georgia State University, Atlanta, GA 30302, USA; ezafar@outlook.com (Z.I.); mahfuz.gsu@gmail.com (M.M.R.); qzia1@student.gsu.edu (Q.Z.); ppopov1@gsu.edu (P.P.); zfu@gsu.com (Z.F.); vcalhoun@gsu.edu (V.D.C.); 2 Center for Translational Research in Neuroimaging and Data Science (TReNDS), Atlanta, GA 30303, USA

**Keywords:** interpretability, explainability, schizophrenia, Alzheimer’s disease, autism spectrum disorder, fMRI, pretraining, latent features, self-supervised, time reversal

## Abstract

Objective: This study aims to develop and validate an interpretable deep learning framework that leverages self-supervised time reversal (TR) pretraining to identify consistent, biologically plausible functional network biomarkers across multiple neurological and psychiatric disorders. Methods: We pretrained a hierarchical LSTM model using a TR pretext task on the Human Connectome Project (HCP) dataset. The pretrained weights were transferred to downstream classification tasks on five clinical datasets (FBIRN, BSNIP, ADNI, OASIS, and ABIDE) spanning schizophrenia, Alzheimer’s disease, and autism spectrum disorder. After fine-tuning, we extracted latent features and employed a logistic regression probing analysis to decode class-specific functional network contributions. Models trained from scratch without pretraining served as a baseline. Statistical tests (one-sample and two-sample *t*-tests) were performed on the latent features to assess their discriminative power and consistency. Results: TR pretraining consistently improved classification performance in four out of five datasets, with AUC gains of up to 5.3%, particularly in data-scarce settings. Probing analyses revealed biologically meaningful and consistent patterns: schizophrenia was associated with reduced auditory network activity, Alzheimer’s with disrupted default mode and cerebellar networks, and autism with sensorimotor anomalies. TR-pretrained models produced more statistically significant latent features and demonstrated higher consistency across datasets (e.g., Pearson correlation = 0.9003 for schizophrenia probing vs. −0.67 for non-pretrained). In contrast, non-pretrained models showed unstable performance and inconsistent feature importance. Conclusions: Time Reversal pretraining enhances both the performance and interpretability of deep learning models for fMRI classification. By enabling more stable and biologically plausible representations, TR pretraining supports clinically relevant insights into disorder-specific network disruptions. This study demonstrates the utility of interpretable self-supervised models in neuroimaging, offering a promising step toward transparent and trustworthy AI applications in psychiatry.

## 1. Introduction

The human brain is a complex, hierarchical network where billions of neurons organize into specialized circuits and functional regions, with neuronal activation during rest or task states constituting information processing that emerges from spatiotemporal activity patterns linking structure and function [1]. Functionally, the brain is divided into distributed networks that dynamically reconfigure in response to cognitive demands and sensory inputs. Advances in neuroimaging, particularly functional MRI (fMRI), have enabled detailed investigations of these network interactions, spurring significant scientific and medical interest [2,3].

The physiological foundation of fMRI lies in the blood oxygen level-dependent (BOLD) signal, an indirect measure of neuronal activity that reflects changes in blood flow, volume, and oxygenation. Unlike direct neural recordings, the BOLD signal captures slow hemodynamic responses, providing an indirect but spatially precise window into brain function [4]. This allows fMRI to map large-scale functional networks across the entire brain with high anatomical resolution, making it particularly suited for investigating the sustained, network-level dysfunctions that characterize many psychiatric and neurological disorders [5]. The temporal characteristics of these BOLD signals—typically sampled every 1–2 s—capture low-frequency fluctuations (<0.1 Hz) that are fundamental to organizing resting-state brain dynamics [6].

Brain dynamics—captured via fMRI and other modalities like electroencephalography (EEG)—describe how and why functional networks evolve over time, offering critical insights into cognition, behavior, and disease mechanisms [7], while dysregulation of these dynamic processes contributes to neuropsychiatric disorders such as schizophrenia (Sch) and Alzheimer’s disease (AD) where aberrant network flexibility impairs cognitive function [8,9].

These neuroimaging advances have been complemented by developments in machine learning approaches to analyze brain network data. Machine learning has played a significant role in studying psychiatric disorders during the last two decades [10,11]. Traditional techniques like support vector machines and logistic regression depend on carefully designed features and statistical methods to identify patterns in data. While these approaches work well for structured information, they often face difficulties when analyzing complex, high-dimensional fMRI and EEG data because they cannot automatically discover meaningful patterns across different levels of abstraction. This challenge led researchers to explore deep learning methods that can automatically learn hierarchical features from raw neuroimaging data through multiple processing layers [12]. These advanced techniques have demonstrated particular success in identifying subtle patterns in brain activity without requiring manual feature selection, with studies consistently showing deep learning’s advantages for analyzing complex brain data [12,13,14].

The application of these machine learning methods has been extensively studied in electrophysiological modalities like electroencephalography (EEG) and event-related potentials (ERPs). EEG provides direct measurement of neuronal electrical activity with millisecond temporal resolution, ideal for capturing rapid neural dynamics and transient cognitive processes. In contrast, fMRI offers superior spatial resolution and is uniquely capable of visualizing deep subcortical structures, making it better suited for investigating sustained, network-level functional integration across the entire brain. While EEG analytics often focus on spectral power, coherence, or transient evoked responses, fMRI analysis typically characterizes low-frequency BOLD signal correlations and their temporal dynamics. This fundamental difference in physiological origin and spatiotemporal resolution means that while the machine learning paradigms may be similar, the informative characteristics they extract—and thus their clinical interpretations—are modality-specific [15].

While these deep learning approaches show superior performance, their “black box” nature limits clinical adoption [16]. Deep learning models employ an end-to-end training approach where neural networks composed of multiple interconnected layers—such as convolutional neural network–long short-term memory (CNN-LSTM) architectures—learn complex, nonlinear relationships in the data. However, due to their intricate architecture, it is highly challenging to fully comprehend how these models arrive at specific decisions, making deep learning inherently opaque [17]. This opacity is particularly problematic in healthcare, where transparency is critical for clinical trust and adoption. To address this, Explainable AI (XAI) has emerged as a field dedicated to developing techniques that enhance interpretability while maintaining model performance [18].

XAI methods can be categorized along three primary dimensions: model-based versus post hoc, model-specific versus model-agnostic, and global versus local explanations [17]. These XAI approaches have been increasingly applied to neuroimaging deep learning models to provide clinically meaningful explanations. For example, [19] applied Integrated Gradients on an LSTM-attention model to generate post-hoc explanations, while [20] proposed Retain And Retrain to validate mental disorder biomarkers. Other approaches like constrained source-based salience have combined active subspace learning with spatially constrained independent component analysis (ICA) to identify AD-related gray matter changes [21]. However, saliency-based methods exhibit several limitations: they lack sensitivity to internal model representations, focus on input-output correlations that may not reflect causal mechanisms, and often produce noisy attribution patterns [22,23].

Given these limitations, probing offers particular promise for interpreting fMRI-based deep learning models by directly interrogating latent representations through controlled experiments. Self-supervised pre-training has emerged as a powerful strategy for learning generalizable representations from unlabeled fMRI or EEG data, with various pretext tasks proposed, including contrastive learning [24] and masked autoencoding [25]. One such method, order-contrastive pretraining [26], involves sampling pairs of time segments to learn temporal dynamics. In this work, we build upon this foundation by employing a time-reversal classification task [19,27], a self-supervised objective designed to learn robust spatiotemporal features. We adapt the wholeMILC architecture [28], a CNN-LSTM model pretrained via this time-reversal method, to analyze seven functional networks across three neurological conditions: schizophrenia, AD, and autism spectrum disorder. By training simple classifiers (e.g., linear models) on the intermediate latent features from this adapted model, our probing analysis provides quantitative measures of feature encoding, revealing what discriminative information is present in the learned representations [29] to elucidate the model’s decision-making process.

Our approach interprets model decisions by (1) extracting latent features, (2) probing them with logistic regression, and (3) analyzing feature-prediction relationships. By mapping long short-term memory (LSTM) and attention weights to functional networks, we identify network-specific alterations across all three conditions compared to healthy controls. In summary, this work advances neuroimaging analysis by transforming latent representations into interpretable features, enabling new insights into functional connectivity changes across neurological and neurodevelopmental disorders.

## 2. Materials and Methods

Our proposed methodology consists of four phases. First, the model undergoes pretraining with time-reversed inputs to learn robust temporal representations. Next, we perform downstream classification training using transfer learning to adapt the pretrained model to specific tasks. We then learn a mapping from the model’s raw inputs to its latent feature representations. Finally, we probe the latent features by fitting a logistic regression classifier and analyzing the coefficients to determine feature importance for each class. The most influential features are mapped back to functional networks in the input space to interpret their biological or functional relevance.

### 2.1. Pretraining Method

We employ our previously proposed pretraining method called Time Reversal (TR), whose efficacy in improving model performance and interpretability has been rigorously demonstrated in [19,27]. In this paper, we briefly summarize this self-supervised pretraining scheme.

We use ICA-preprocessed fMRI data, which consists of spatial components (capturing brain network activations) and time points (representing temporal dynamics). In our pretraining method, we artificially augment the dataset by reversing the temporal order of the time series for each component, effectively doubling the dataset size—half original and half time-reversed. The model is then pretrained to distinguish between original and time-reversed sequences. By learning to detect the direction of time, the model captures essential fMRI signal dynamics, which enhances its performance in downstream tasks on related datasets.

#### Mathematical Description of Time Reversal

Let $X=\{x_{1},x_{2},\ldots ,x_{T}\}$ be an fMRI time series for a given component, where $x_{t}\in R^{d}$ represents the brain activity at time *t*. The time-reversed version is constructed as $\tilde{X}=\left\{x_{T},x_{T-1},\ldots ,x_{1}\right\}$. The pretraining task involves training a model $f_{\theta }$ to classify whether an input sequence *X* is original (y=0) or reversed (y=1), optimizing:

(1) $$L=-E_{X∼D}\left[y\log\left(f_{\theta }\left(\tilde{X}\right)\right)+\left(1-y\right)\log\left(f_{\theta }\left(X\right)\right)\right]$$

where D is the original data distribution.

A key advantage of this method is its data efficiency—it enables reliable performance even with limited fMRI datasets, mitigating the challenge of data scarcity in healthcare applications.

### 2.2. Datasets

We conducted experiments using six distinct fMRI datasets. All datasets were preprocessed using the pipeline described in Section 2.3. For pretraining, we utilized the Human Connectome Project (HCP) dataset [30] consisting of 823 healthy control (HC) subjects. Each subject’s data comprised 53 non-noise components (extracted through our preprocessing pipeline) with 1200 time points per component.

The time reversal pretraining objective enables the model to learn generalizable temporal dynamics and signal patterns across the time series. This pretrained knowledge facilitates faster convergence in downstream classification tasks, particularly beneficial in low-data scenarios.

For downstream evaluation, we employed five additional datasets representing three psychiatric conditions:

- Schizophrenia (Sch) (2 datasets);
- Alzheimer’s Disease (AD) (2 datasets);
- Autism Spectrum Disorder (ASD) (1 dataset).

#### 2.2.1. Schizophrenia

Schizophrenia is a severe psychiatric disorder characterized by progressive deterioration of brain function, manifesting through symptoms including delusions, thought disorder, hallucinations, motor and cognitive impairments, and diminished emotional expression [31]. Recognized as one of humanity’s most debilitating health conditions [32], Sch remains fundamentally enigmatic despite significant neuroscientific advances and extensive research efforts [33]. Early detection of potential Sch spectrum disorders is crucial for timely intervention and effective management of psychotic symptoms [34], highlighting the need for biomarkers.

For our downstream classification tasks, we employed two well-established Sch datasets: the FBIRN dataset [35] (311 subjects: 151 HCs and 160 patients) and the B-SNIP dataset [36] (589 subjects: 338 HCs and 251 patients). These complementary datasets enable comprehensive evaluation of our model’s ability to identify disease-related patterns across different patient cohorts and imaging protocols.

#### 2.2.2. Alzheimer’s Disease

AD is a progressive neurological disorder and the most common form of dementia, characterized by declining memory function and deterioration of cognitive, behavioral, and social functioning over time. Known risk factors include advanced age, genetic predisposition, and traumatic brain injury [37]. The insidious progression of cognitive decline underscores the critical need for early detection methods.

For AD classification, we utilized two comprehensive datasets: the OASIS dataset [38] (823 subjects: 651 HCs and 172 patients) and the ADNI dataset [39] (499 subjects: 433 HCs and 66 patients). These datasets provide complementary perspectives on AD progression, with OASIS offering broader population representation and ADNI providing detailed longitudinal tracking of disease progression.

#### 2.2.3. Autism Spectrum Disorder

ASD is a neurodevelopmental condition characterized by persistent deficits in social communication and interaction, as defined by the DSM-5 criteria. These deficits manifest through: (1) impaired social-emotional reciprocity, (2) deficits in nonverbal communicative behaviors, and (3) difficulties in developing and maintaining relationships. Additionally, individuals with ASD exhibit restricted, repetitive patterns of behavior, interests, or activities, which may include stereotyped movements, rigid routines, or sensory abnormalities [40]. While symptoms emerge during early development, they may only become fully apparent after the second year of life, often causing significant functional impairment across social and occupational domains.

The etiology of ASD remains largely idiopathic, though approximately 10–20% of cases can be attributed to identifiable genetic factors such as fragile X syndrome or tuberous sclerosis [41]. For our analysis, we utilized the ABIDE dataset [42], which comprises 869 subjects (398 HCs and 471 individuals with ASD), providing a well-balanced sample for investigating neural signatures of the disorder.

Despite their apparent differences, Sch, ASD, and AD share several clinically meaningful features. All three conditions can demonstrate deteriorating courses, though this progression is most characteristic of AD and certain subtypes of Sch and ASD. These disorders exhibit overlapping symptom domains, including positive symptoms (e.g., delusions and hallucinations) that hallmark Sch but may also emerge in AD and some ASD presentations. Notably, they also share negative symptoms such as emotional blunting, motivational impairment, and reduced speech output—ranging from poverty of speech in Sch to complete mutism in severe ASD cases, late-stage AD, and during acute schizophrenic episodes with catatonic features [40]. This clinical overlap suggests potential common pathways in neural dysfunction that warrant cross-disorder investigation.

All primary data utilized in this study were acquired using blood oxygen level-dependent (BOLD) functional MRI (fMRI), the standard methodology for assessing brain activity non-invasively by measuring hemodynamic changes linked to neural firing. This is the dominant contrast mechanism for fMRI research. The datasets—HCP, FBIRN, BSNIP, ADNI, OASIS, and ABIDE—all share this fundamental basis, ensuring the analyzed signals reflect the same underlying neurovascular coupling processes. While acquisition parameters (e.g., repetition time, scanner field strength) varied across consortia, the core BOLD fMRI technique provides a consistent foundation for investigating functional connectivity and brain dynamics across the studied neurological and psychiatric conditions. The datasets encompassed a wide range of ages and clinical conditions, as detailed in Table 1.

### 2.3. Preprocessing

The resting-state fMRI data preprocessing was performed using Statistical Parametric Mapping (SPM12) in MATLAB 2016. The first five scans were discarded to allow for signal equilibrium and scanner noise adaptation, followed by motion correction using SPM’s rigid body transformation and slice-timing correction to account for temporal differences in slice acquisition. The fMRI data were then normalized to the Montreal Neurological Institute (MNI) space using an echo-planar imaging (EPI) template, resampled to 3×3×3 mm^3^ voxel size, and smoothed with a 6 mm full width at half maximum (FWHM) Gaussian kernel. Subjects exhibiting head motion exceeding $3^{\circ }$ of rotation or 3 mm of translation were excluded from further analysis. Rigorous quality control was implemented, including visual inspection of normalization results and exclusion of subjects with poor brain coverage. For component extraction, we employed a fully automated framework using ICA, which provides more robust functional representations than anatomical parcellations. Spatial group ICA was conducted on two independent datasets (HCP and Genomics Superstruct Project) to generate network templates, with components matched across datasets using a spatial correlation threshold of ≥0.4. A subset of matched components was classified as intrinsic connectivity networks (ICNs) through expert consensus (five fMRI researchers), evaluated based on three criteria: gray matter activation peaks, minimal overlap with vascular/ventricular/motion artifacts, and dominant low-frequency fluctuations. Components receiving ≥3 expert votes were retained as meaningful ICNs, resulting in 53 well-matched intrinsic networks that were used for subsequent analysis [20,43,44].

### 2.4. Data Balancing Strategy

The OASIS and ADNI datasets exhibit significant class imbalance, with HCs substantially outnumbering AD patients. For OASIS, the original distribution comprises 651 HCs and 172 AD subjects, while ADNI contains 433 HCs and 66 AD subjects. To address this imbalance, we developed a data balancing method that strategically selects a subset of HC subjects whose functional connectivity patterns most closely resemble the AD population. First, we extracted subject-level embeddings by averaging each subject’s fMRI time series across time points, resulting in 53-dimensional feature vectors (one per ICA component). Using Mahalanobis distance, we quantified the similarity between each HC subject’s embedding and the AD group distribution. For ADNI, we retained the 100 HC subjects closest to the AD centroid, and for OASIS, we selected the top 261 HC subjects. This approach preserves data-driven biological relevance while balancing class sizes (100 HC/66 AD for ADNI; 261 HC/172 AD for OASIS). The selected HC subsets were combined with all available AD subjects, followed by randomization.

All other datasets in our study (FBIRN, B-SNIP, and the autism dataset) were naturally balanced or required only simple random subsampling to achieve parity between classes. The Mahalanobis-based balancing was uniquely applied to OASIS and ADNI due to their pronounced imbalance (HC:AD ratios of 3.8:1 and 6.6:1, respectively), which could otherwise bias model training. This method ensures representative sampling while maintaining the integrity of the underlying neurobiological relationships.

### 2.5. Training Methodology

Our proposed methodology comprises four key phases designed to identify disorder-specific functional connectivity disruptions. First, we pretrain an LSTM model on resting-state fMRI data from HCs in the HCP dataset (823 subjects), using a time-reversal pretext task. This self-supervised approach forces the model to learn generalizable temporal dynamics by distinguishing between original and time-reversed fMRI sequences, capturing essential patterns of brain signal propagation without requiring labeled clinical data.

For downstream analysis, we transfer these pretrained weights to classification tasks across five independent datasets spanning three neurological conditions: Sch (FBIRN, B-SNIP), AD (OASIS, ADNI), and ASD (ABIDE). The pre-trained model provides a powerful initialization for downstream tasks by supplying a set of general-purpose features that encode fundamental temporal dynamics of brain activity. These features, which capture how neural signals evolve in time, are highly transferable as they represent a foundational layer of brain function upon which disease-specific perturbations can be more easily detected. Fine-tuning then efficiently adapts these general features to the specific diagnostic task to highlight the disruptions in these dynamics that are most indicative of a particular disorder.

The model processes windowed fMRI inputs (20 time points/window) through a hierarchical architecture with dual attention mechanisms—first at the window level to localize temporal features, then globally to weight informative windows. The window size was determined through empirical optimization to capture meaningful temporal dynamics without sacrificing resolution. We evaluated a range of window sizes (10, 20, 40, and 80 time points) on a held-out validation subset of the HCP pre-training data. A window size of 20 time points consistently yielded the highest accuracy on the time-reversal pretext task, suggesting it provides an optimal balance for the model to learn discriminative short- to medium-range temporal dependencies in fMRI data. We extract latent feature representations (200-dimensional vectors) from the final attention layer, which encode the model’s distilled understanding of each subject’s functional connectivity patterns.

To interpret these learned representations, we employ probing via logistic regression, where latent features predict diagnostic labels (HC vs. patient). By analyzing the regression coefficients, we quantify feature importance for each disorder. Positive weights indicate features associated with pathology, while negative weights correlate with healthy patterns. These importance scores are then mapped backward through our framework: (1) from logistic regression coefficients to latent features, (2) from latent features to ICA components via the model’s attention weights, and (3) from components to established functional networks (e.g., default mode network, salience network) using predefined neuroanatomical mappings.

**Figure 1:**
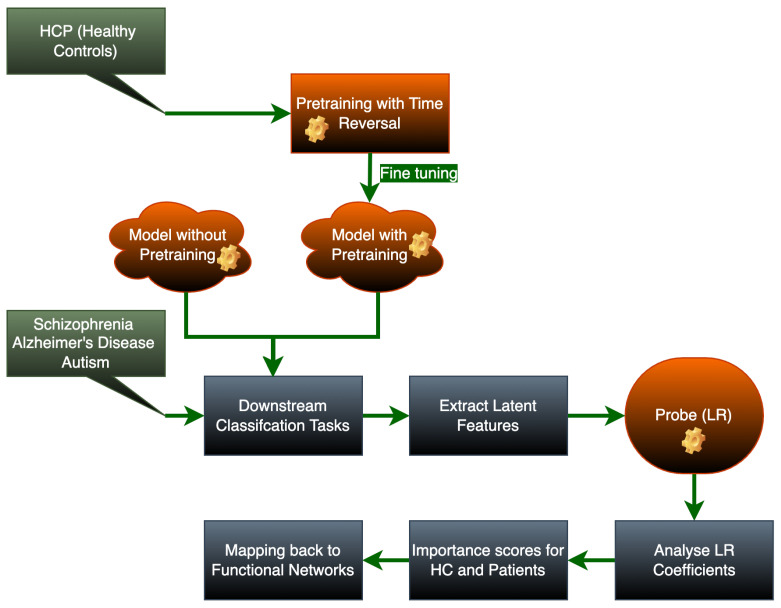
Analytical pipeline for identifying disorder-specific functional connectivity biomarkers: The process begins with self-supervised pretraining on HC data from HCP using time-reversal augmentation to learn normative brain dynamics, followed by transfer learning to downstream classification tasks across three psychiatric disorders (Sch, AD, and ASD). The model’s performance is compared between pretrained and non-pretrained versions at each stage. Finally, latent features extracted from the trained model are analyzed through logistic regression (LR) probing, where importance scores derived from LR coefficients are mapped back to functional networks to reveal disease-specific connectivity patterns (e.g., altered network activity in patients versus HCs).

This pipeline ultimately reveals which functional networks exhibit statistically significant disruption in each condition. The derived biomarkers provide clinically interpretable insights into the neural mechanisms of each disease, validated through comparison with known pathophysiological literature. A schematic description of our proposed methodology is shown in Figure 1.

### 2.6. Model Architecture

**Figure 2:**
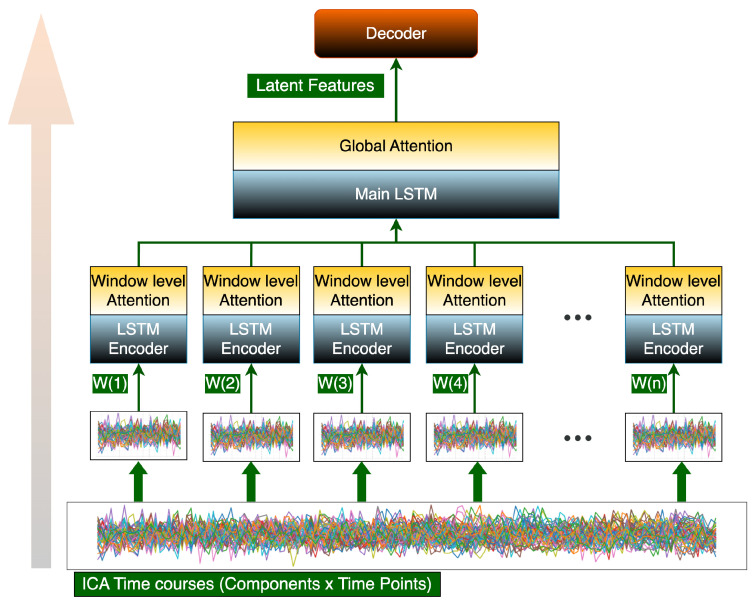
Hierarchical LSTM Architecture with Dual Attention Mechanisms: The model processes ICA time courses through sequential windows (W(1), W(2), …, W(n)). Each window is encoded by an LSTM encoder and weighted via window-level attention. Processed windows are integrated by the main LSTM with global attention, producing latent features for the decoder. Arrows indicate flow direction.

The proposed framework builds upon the whole MILC architecture [28] but replaces the original CNN-based encoder with an LSTM-based encoder, as we empirically found LSTMs to provide more stable and interpretable feature extraction for fMRI time-series data. Figure 2 shows our hierarchical LSTM architecture for both pretraining and downstream classification. It processes fMRI data through a hierarchical LSTM architecture with dual attention mechanisms. The input time series are first divided into fixed-length windows (20 time points per window), with each window processed independently by a unidirectional LSTM encoder (input_size=53, hidden_size=256) that captures local temporal patterns. A window-level attention mechanism then computes weighted combinations of the encoded time points, producing compact window embeddings that emphasize diagnostically relevant segments. These window embeddings are sequentially processed by a global LSTM layer (hidden_size=200) followed by a second attention layer that identifies clinically significant windows across the entire scan. The resulting 200-dimensional latent feature vector z serves dual purposes: (1) as input to a sigmoid-activated decoder for classification (healthy vs. disorder), and (2) as the basis for probing analyses via logistic regression. During pretraining, the model learns normative brain dynamics by classifying time-reversed versus original fMRI sequences from HCs (HCP dataset), while downstream fine-tuning adapts these learned features to specific disorders (Sch, AD, and ASD) through diagnostic label prediction. The attention weights at both levels provide inherent interpretability by highlighting which temporal segments and functional networks contribute most to the model’s decisions.

**Figure 3:**
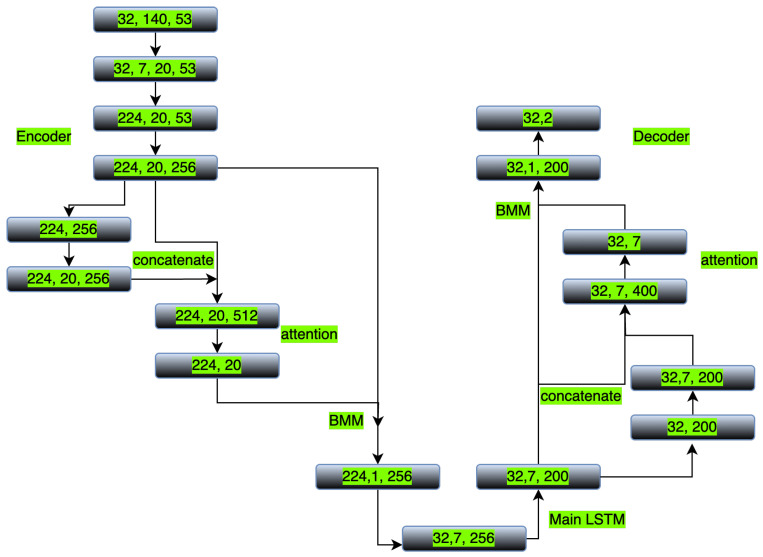
Model architecture with dimensional flow for FBIRN: The input (32 × 140 × 53: batch × time × components) is partitioned into 7 segments (32 × 7 × 20 × 53). Each segment’s data (20 × 53) is encoded by the LSTM to 20 × 256 embeddings, resulting in 32 × 7 × 20 × 256 (reshaped to 224 × 20 × 256 for batched processing). Window-level attention computes weights (224 × 20). These are applied to the embeddings via BMM, an operation that performs a weighted sum across the time dimension, to produce a single attended feature vector per window (224 × 1 × 256), which is reshaped to 32 × 7 × 256. The main LSTM processes this sequence to 32 × 7 × 200. Global attention (weights: 32 × 7) then uses BMM to yield the final latent representation (32 × 1 × 200), which is decoded to predictions (32 × 2). This visualization clarifies how hierarchical attention condenses information at multiple temporal scales while preserving the dimensional integrity critical for interpretability.

Figure 3 illustrates the dimensional flow of the FBIRN data through our hierarchical architecture. The input (32 × 140 × 53), representing batch size, time points, and components, is first windowed into 7 segments (32 × 7 × 20 × 53). Each segment is encoded by an LSTM, producing a sequence of embeddings for each window (32 × 7 × 20 × 256). This tensor is reshaped to (224 × 20 × 256) for batched processing, where 224 is the total number of windows in the batch (32 × 7). The window-level attention mechanism (left section) then computes a weight vector of size (224 × 20) for the 20 time steps in each window. To apply these weights, we employ a batch matrix multiplication (BMM) operation. The attention weights are unsqueezed to (224 × 1 × 20) and multiplied with the value matrix (224 × 20 × 256). This BMM calculates a weighted sum of the time-step vectors, condensing each window into a single 256-dimensional attended representation (224 × 1 × 256), which is reshaped to (32 × 7 × 256). The global attention mechanism (right section) processes this output through the main LSTM to (32 × 7 × 200), where a second BMM operation, using weights of (32 × 7), produces a final subject-level latent representation (32 × 1 × 200) for decoding. This visualization clarifies how both attention layers—operating at different temporal scales via the BMM operation—contribute to the model’s interpretability by highlighting salient windows and time points.

### 2.7. Mapping: Component → Functional Network → Latent Features

To connect the model’s architecture to its interpretability, we developed a backward engineering approach that utilizes the attention weights and LSTM parameters described in Section 2.6. This method leverages the global and temporal attention weights from the fine-tuned model to trace the contribution of each input component to the final latent representations.

**Figure 4:**
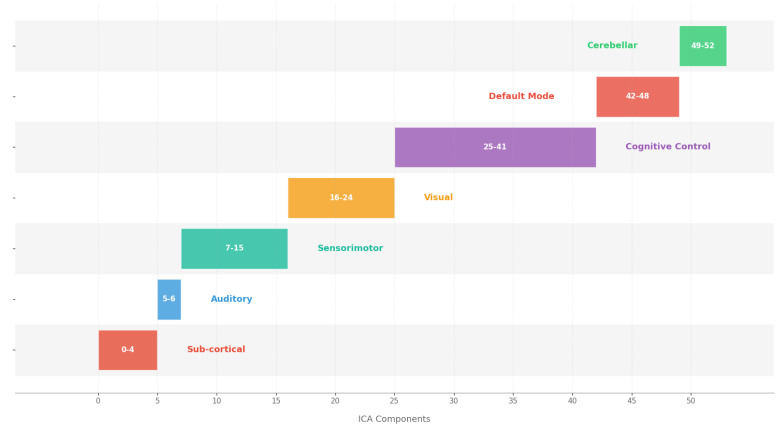
Functional network to ICA component mapping: This visualization maps seven brain functional networks (Default Mode, Cognitive Control, Sensorimotor, Subcortical, etc.) to their constituent ICA components. The table structure shows the component indices (0–53) associated with each network.

Starting with the known mapping between ICA components and functional networks (Figure 4), we first scale the main LSTM weights by their corresponding global attention scores to capture window-level importance. We then scale the encoder weights by their time-step attention scores within each window. By multiplying these two scaled matrices, we obtain a final mapping matrix of dimensions (latent features × components). This matrix enables us to trace how each input component influences the latent representations. A formal description of this process is provided in Algorithm 1.
**Algorithm 1** Attention-Weighted Component-to-Latent Mapping**Notation:**         *C*:                Number of input components (e.g., 53)         $D_{\mathrm{enc}}$:            Encoder LSTM hidden size (e.g., 256)         $D_{\mathrm{main}}$:          Main LSTM hidden size (e.g., 200)         *K*:                Number of windows (e.g., 7)         *T*:                Number of time steps per window (e.g., 20)**Require:**       $W_{\mathrm{enc}}$: Encoder weight matrix $\in R^{D_{\mathrm{enc}}\times C}$       $W_{\mathrm{main}}$: Main LSTM weight matrix $\in R^{D_{\mathrm{main}}\times D_{\mathrm{enc}}}$       $\alpha_{\mathrm{win}}$: Window-level attention matrix $\in R^{K\times T}$       $\alpha_{\mathrm{global}}$: Global attention vector $\in R^{K}$**Ensure:**       *M*: Component-to-latent mapping matrix $\in R^{D_{\mathrm{main}}\times C}$  1:   **procedure**
ComponentToLatentMapping($W_{\mathrm{enc}},W_{\mathrm{main}},\alpha_{\mathrm{win}},\alpha_{\mathrm{global}}$) 2:        M←0                                                                                     ▹ Initialize *M* to zeros 3:        **for** k←1 to *K*
**do**                                                         ▹ Iterate over each window 4:               $W_{\mathrm{main}}^{\left(\mathrm{scaled}\right)}\leftarrow \alpha_{\mathrm{global}}\left[k\right]\cdot W_{\mathrm{main}}$                                ▹ Scale by global importance 5:               **for** t←1 to *T*
**do**                          ▹ Iterate over each time step in window *k* 6:                      $W_{\mathrm{enc}}^{\left(\mathrm{scaled}\right)}\leftarrow \alpha_{\mathrm{win}}\left[k,t\right]\cdot W_{\mathrm{enc}}$                     ▹ Scale by temporal importance 7:                      $\mathrm{contribution}\leftarrow W_{\mathrm{main}}^{\left(\mathrm{scaled}\right)}\times W_{\mathrm{enc}}^{\left(\mathrm{scaled}\right)}$         ▹ Compute transformation path 8:                      M←M+contribution                    ▹ Accumulate weighted contribution 9:             **end for**10:        **end for**11:        **return** *M*12:   **end procedure**

### 2.8. T-Test Statistical Significance

The latent features extracted from the LSTM model undergo two key statistical tests before being used in further probing analysis. First, a one-sample *t*-test is performed to determine whether the features significantly differ from zero across all samples. This test evaluates whether the latent dimensions contain meaningful signal rather than random noise, as features that do not deviate from zero may lack interpretable patterns and could be excluded from downstream analysis. The p-values from this test are corrected for multiple comparisons using the False Discovery Rate (FDR) method to control the likelihood of false positives.

Next, a two-sample *t*-test is conducted to assess whether the latent features exhibit significant differences between each clinical group (Sch, ASD, AD) and HCs. This test identifies which features are discriminative of the clinical conditions, suggesting their potential relevance as biomarkers. As with the one-sample test, FDR correction is applied to account for multiple hypothesis testing.

The significance threshold is set at *p* < 0.05, a conventional benchmark in statistical hypothesis testing that balances sensitivity and specificity. This threshold implies a 5% risk of falsely rejecting the null hypothesis (Type I error), which is widely accepted in biomedical and computational research. While arbitrary, this cutoff provides a standardized criterion for identifying statistically meaningful effects while maintaining reasonable statistical power. By applying this threshold after FDR correction, we further reduce the likelihood of spurious findings, ensuring that only robustly significant features are retained for probing.

These tests collectively serve as a quality control step, verifying that the latent features carry meaningful signal and exhibit clinically relevant group differences before being used in subsequent analyses. This approach strengthens the validity of any conclusions drawn from the model’s representations and ensures that downstream interpretations are based on statistically reliable patterns.

### 2.9. Probing Analysis of Latent Features

To interpret the latent features learned by the model, we employed a probing analysis framework. This involved training a linear logistic regression classifier on the extracted latent representations to perform diagnostic classification. The coefficients of this probe classifier were then analyzed to determine the importance of each latent feature for distinguishing between clinical groups and healthy controls. A feature’s coefficient magnitude indicates its overall contribution to the decision, while its sign indicates class association—positive values being more predictive of the healthy control group and negative values more predictive of the patient group. This probing procedure was applied consistently across models fine-tuned for schizophrenia, Alzheimer’s disease, and autism spectrum disorder, allowing for a comparative analysis of discriminative features and potential biomarkers across multiple neurological and psychiatric conditions.

### 2.10. Implementation Details

To ensure full reproducibility, we provide a comprehensive description of our computational setup. All models were implemented in PyTorch (https://pytorch.org/, accessed on 28 August 2025) and trained on NVIDIA DGX nodes equipped with V100 GPUs (8 per node, 40 GB VRAM each) and 512 GB of system memory. For both pre-training on HCP and downstream fine-tuning, we used a batch size of 32 and the Adam optimizer with a learning rate of $7\times 10^{-4}$. We employed early stopping with a patience of 50 epochs and a minimum delta threshold of 0.0001 to determine convergence. Regarding computational efficiency, we observed that models initialized with pre-trained weights converged significantly faster than those trained from scratch, often requiring fewer epochs and less wall-clock time to reach optimal performance on the downstream task. This demonstrates that the pre-training phase not only improves accuracy but also enhances computational efficiency.

## 3. Results

Our results span five datasets across three psychiatric conditions (Sch, AD, and ASD), evaluated through a multi-stage validation framework. We first pretrained five model variants on the HCP HC dataset using five-fold cross-validation, with each fold yielding a distinct pretrained model. For downstream analysis, each pretrained model was evaluated on target datasets using four random seeds, resulting in 100 total experiments (5 pretrained models × 5 dataset folds × 4 seeds). Latent features from these experiments were processed through our mapping pipeline (Section 2.7), where logistic regression probes—themselves evaluated with five-fold cross-validation—generated class-specific importance scores. These scores were mapped back to functional networks to identify consistent disorder-related connectivity patterns. All reported figures adhere to this protocol, ensuring robust and reproducible comparisons across conditions.

**Figure 5:**
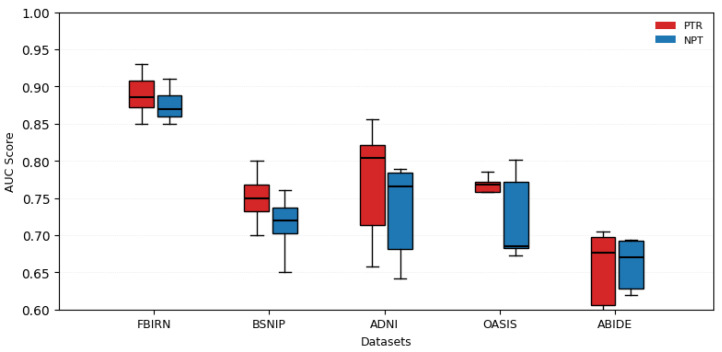
Comparison of AUC Scores with and without Pretraining (PTR vs. NPT) Across Five Datasets: The comparison of AUC scores between pretrained (PTR) and non-pretrained (NPT) models reveals distinct patterns across datasets. Pretraining yields consistent improvements in four datasets: BSNIP shows a 5.3% increase (PTR 0.750 vs. NPT 0.712), ADNI a 5.2% gain (0.770 vs. 0.732), OASIS a 3.5% improvement (0.747 vs. 0.722), and FBIRN a more modest 2.4% uplift (0.885 vs. 0.864). In contrast, ABIDE demonstrates comparable performance between PTR (0.653) and NPT (0.661), likely due to its larger sample size (869 subjects), which provides sufficient training data even without pretraining. This divergence highlights that while pretraining generally enhances performance for smaller datasets, its benefits diminish when ample training data is available.

The results of the downstream classification tasks, evaluated using AUC scores, demonstrate the effectiveness of pretraining across five datasets: FBIRN, BSNIP, ADNI, OASIS, and ABIDE. Figure 5 compares the performance of models trained with pretraining (PTR) and without pretraining (NPT). The findings reveal a consistent improvement in AUC scores for the PTR models, indicating that pretraining enhances the model’s ability to generalize and perform well on downstream tasks. This suggests that the features learned during pretraining are transferable and beneficial across diverse datasets.

The statistical validation of the 200-dimensional latent features reveals that pretraining yields more meaningful representations. As shown in Table 2, features extracted using pretrained weights exhibit significantly stronger group differences (two-sample *t*-test, FDR-corrected *p* < 0.05) and greater deviation from zero (one-sample *t*-test, FDR-corrected *p* < 0.05) compared to those from non-pretrained models. This aligns with the downstream classification results, where pretraining improved AUC scores by 2.4–5.3% across most datasets, confirming that pretrained features are both statistically robust and clinically discriminative.

The results of the probing analysis are shown in the subsequent figures. In all figures, the *x*-axis represents the functional brain networks, while the *y*-axis indicates the weighted mean coefficient value, which reflects the importance of each network in distinguishing between patients and healthy controls. The analysis was based on a substantial sample size of 100 bootstrap iterations per dataset (derived from 5 models × 5 folds × 4 seeds). Error bars are included to capture the variability across cross-validation folds, providing insight into the stability of the model’s interpretations.

A positive mean coefficient for a functional network indicates that its activity patterns are more representative of healthy controls, suggesting preserved or typical functioning within that network. In contrast, a negative coefficient implies that the network contributes more strongly to identifying individuals with the disorder, reflecting potential disruption, dysregulation, or disease-related alterations. Thus, networks with negative coefficients may serve as candidate biomarkers of pathological changes, while those with positive coefficients may highlight regions where normal functional integrity is maintained.

**Figure 6:**
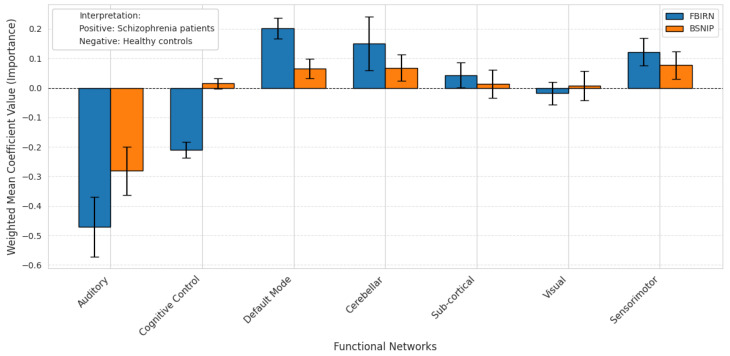
Probing analysis using pretrained model weights on the FBIRN (blue) and BSNIP (orange) datasets for Sch. The Auditory network consistently shows negative coefficients with low variability across folds in both datasets, aligning with known disruptions in auditory processing in Sch. The Cognitive Control network exhibits a negative coefficient in FBIRN but a slightly positive one in BSNIP. Default Mode, Sensorimotor, and Cerebellar networks display positive coefficients, suggesting preserved or compensatory roles. Subcortical and Visual networks show high variability, with the Visual network presenting opposite coefficient signs across datasets. This divergence may be due to differences in resting-state acquisition protocols—FBIRN employed an eyes-closed setup, while BSNIP used eyes-open.

**Figure 7:**
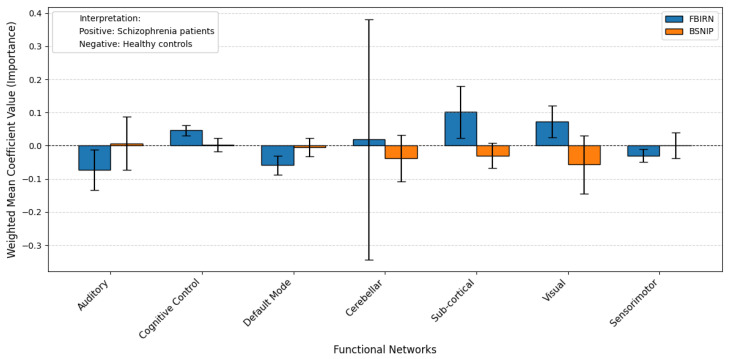
Probing analysis of Sch using non-pretrained model weights on FBIRN (blue) and BSNIP (orange) datasets: Compared to the pretrained case in Figure 6, results here show increased variability and reduced alignment between the two datasets, as reflected by a Pearson correlation of −0.67. The direction of association for several networks (e.g., Default Mode, Cerebellar, Subcortical) varies notably between datasets, suggesting instability and reduced reliability in identifying consistent functional biomarkers without pretraining.

Figure 6 and Figure 7 show results for Sch classification using two datasets: FBIRN and BSNIP. Figure 6 presents the results when TR pretraining was used, while Figure 7 shows the results without pretraining. The probing analysis reveals that when pretrained weights are used, the variability across folds is considerably lower, indicating greater stability and consistency in model interpretations. This is further supported by the Pearson correlation coefficient between the coefficient vectors of FBIRN and BSNIP, which is 0.9003 in the pretrained case, suggesting a high degree of alignment in how the model evaluates functional network importance across datasets.

For example, in Figure 6, the Auditory network consistently shows negative coefficients with low variability for both datasets, aligning with known auditory processing disruptions in Sch. The Cognitive Control network shows a negative coefficient for FBIRN but a small positive coefficient for BSNIP. Networks such as the Default Mode, Sensorimotor, and Cerebellar show positive coefficients, while the Sub-cortical and Visual networks display higher variability. In the Visual network, the opposite signs across datasets may be attributed to the difference in resting-state acquisition protocols—FBIRN used an eyes-closed protocol, while BSNIP used eyes-open.

In contrast, the results in Figure 7 (non-pretrained) demonstrate a lack of consistency. The Pearson correlation drops to −0.67, indicating that the model prioritizes different networks for the same condition across datasets. Many networks also show opposite coefficient signs and significantly larger error bars, pointing to increased variability and reduced reliability when pretraining is not used.

**Figure 8:**
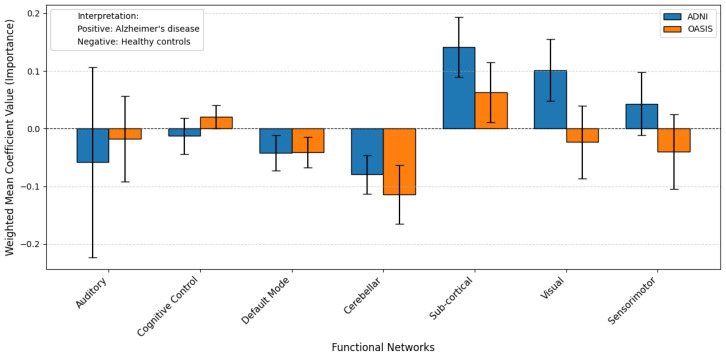
Weighted mean coefficient values from probing analysis using pretrained model weights for AD classification on ADNI (blue) and OASIS (orange) datasets: A general agreement is observed between the two datasets across most networks, particularly Subcortical, Default mode, and Cerebellar. The Pearson correlation between the two datasets is 0.66. This alignment supports the robustness of the pretrained model in identifying consistent neural biomarkers across datasets despite differences in sample collection and population.

**Figure 9:**
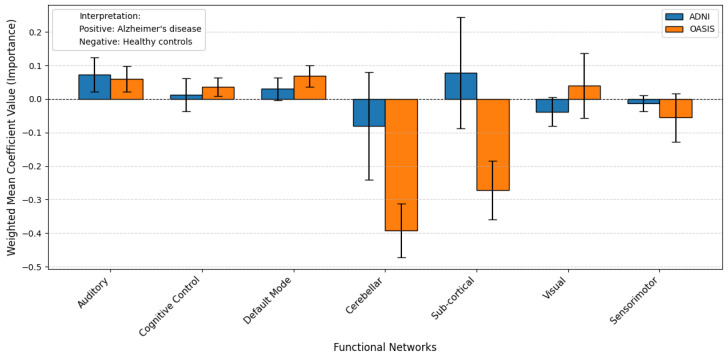
The figure presents weighted mean coefficient values from a probing analysis conducted without pretrained model weights for AD classification, comparing results from the ADNI (blue) and OASIS (orange) datasets. Unlike the pretrained version, which showed strong agreement (Pearson r = 0.66), the correlation here drops to 0.32, indicating reduced consistency between datasets. Notably, several networks exhibit coefficients in opposing directions—where one dataset associates a network with disease (negative), the other links it to controls (positive), or vice versa. This divergence suggests that without pretraining, the model captures dataset-specific noise or biases rather than robust disease-related patterns. The weaker correlation and opposing trends highlight the importance of pretraining in aligning neural biomarkers across diverse cohorts, as untrained models may struggle to generalize findings consistently.

A similar trend is observed for AD, using the ADNI and OASIS datasets. Figure 8 and Figure 9 show the results with and without pretraining, respectively. In the pretrained model (Figure 8), most functional networks exhibit stable coefficients across folds, though the Auditory network shows higher variability. The Cerebellar and Default Mode networks show negative coefficients, while the Sub-cortical network exhibits a positive direction. In some cases, networks display opposite coefficient signs across datasets, which may indicate that the model is unable to confidently generalize across datasets—potentially due to differences in age distribution, scanner protocols, or disease progression stages. The Pearson correlation for pretrained models is 0.66, suggesting moderate alignment, whereas in the non-pretrained case (Figure 9), the correlation drops to 0.32, reinforcing the instability of models trained from scratch.

**Figure 10:**
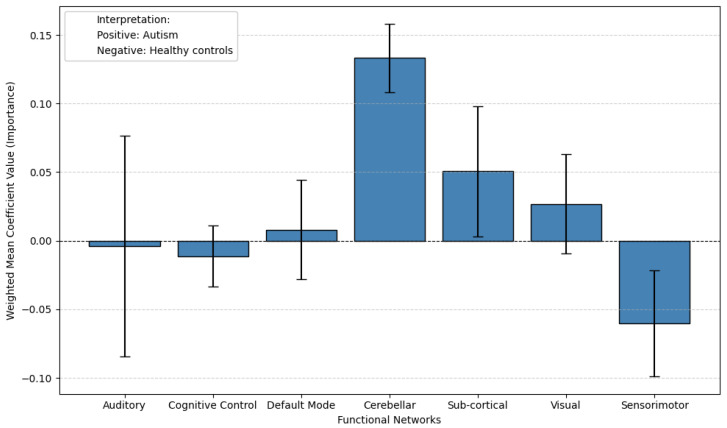
Probing results from the pretrained model on the ABIDE dataset reveal distinct functional network associations with ASD. The Sensorimotor network shows negative coefficients, suggesting its reduced engagement may be characteristic of ASD, while the Cerebellar and Sub-cortical networks exhibit positive coefficients, indicating relatively preserved or compensatory activity in healthy controls. However, other networks (e.g., Default Mode, Cognitive Control) display larger error bars, reflecting substantial variability and making their interpretation less reliable.

**Figure 11:**
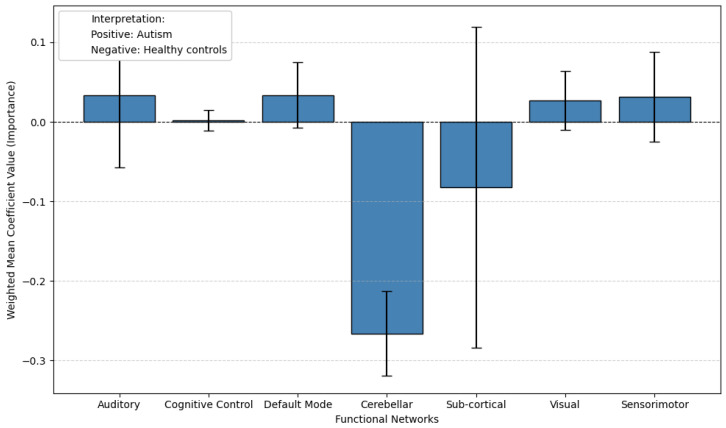
The non-pretrained model’s probing results on the ABIDE dataset reveal substantially different patterns compared to the pretrained version. Most functional networks now show negative coefficients, with the strongest effects appearing in Cerebellar (−0.27). The Cerebellar network, which showed positive association with ASD in the pretrained model, now displays a negative coefficient. This reversal of key network associations suggests the non-pretrained model fails to capture meaningful ASD biomarkers.

For ASD, we used a single dataset, ABIDE, as no comparable second dataset was available. Probing results are shown in Figure 10 (pretrained) and Figure 11 (non-pretrained). In the pretrained setup, the Sensorimotor network has negative coefficients, while the Cerebellar and Sub-cortical networks show positive values. Other networks display larger error bars, making interpretation less reliable due to higher uncertainty. This suggests that while TR pretraining provides some meaningful features, the overall interpretability is limited in this case, possibly due to heterogeneity in the ABIDE dataset. In the non-pretrained model (Figure 11), the Cerebellar network shifts to the negative side, and most other networks show considerable variability, further demonstrating the reduced reliability of models trained from scratch.

In summary, across all conditions—Sch, AD, and ASD—models using TR pretraining consistently exhibit lower variability, higher inter-dataset correlation, and more biologically plausible feature importance patterns compared to non-pretrained models. These findings highlight the critical role of pretraining in improving both performance and interpretability in deep learning models for neuroimaging-based diagnosis.

## 4. Discussion

Functional MRI (fMRI) is a powerful neuroimaging technique used to explore functional connectivity among different brain regions, both during task-specific activities and in resting-state conditions. It is preferred in clinical and research settings due to its non-invasive nature. However, fMRI data is inherently high-dimensional and often contaminated with noise, necessitating rigorous preprocessing before meaningful analysis can be conducted. One commonly used preprocessing technique is ICA, which enables the separation of noise from meaningful signal components in the data.

In recent years, deep learning approaches have been increasingly applied to fMRI data for diagnostic purposes. These models have demonstrated impressive performance; however, their deployment in clinical decision support systems remains limited due to their intrinsic “black-box” nature. This lack of transparency hinders trust in the model’s predictions, making it difficult for clinicians to adopt them in practice. XAI addresses this challenge by aiming to elucidate the model’s decision-making process. Traditional input–output-based techniques, such as saliency maps, often struggle to capture the internal reasoning of the model, leading to noisy and inconsistent attribution patterns. In contrast, probing methods offer a more interpretable approach by analyzing the latent (intermediate) representations learned by the model. These methods help reveal how the model transforms input data into representations that influence the final prediction.

To enhance the consistency and performance of deep learning models in fMRI-based classification tasks, we employ a pretraining method called TR. This technique improves the AUC scores and helps the model produce high-confidence predictions. Importantly, TR pretraining also leads to more stable and reproducible results across different random seeds and cross-validation folds, as demonstrated in the Results Section.

In our study, we first pretrain a model using the TR method on the HCP dataset. The learned weights from this pretraining phase are then transferred to downstream classification tasks involving three different clinical conditions. From these fine-tuned models, we extract the latent features, which are then fed into a linear probe (logistic regression) to analyze the relationship between these features and class labels. By examining the logistic regression coefficients and mapping them back to their corresponding functional brain networks, we identify which networks are most affected in each specific disorder.

To evaluate the impact of TR pretraining, we also train models from scratch for each downstream task—without using the pretrained weights. Our comparison of models with and without TR pretraining across five neuroimaging datasets (FBIRN, BSNIP, ADNI, OASIS, ABIDE) reveals two key findings. First, TR pretraining consistently enhanced classification performance in four datasets, with AUC improvements ranging from 2.4% (FBIRN) to 5.3% (BSNIP), underscoring its value in data-scarce scenarios. Notably, the largest gains occurred in BSNIP (+5.3%) and ADNI (+5.2%), where pretrained models achieved AUCs of 0.750 and 0.770, respectively, compared to 0.712 and 0.732 for non-pretrained models. Second, the exception—ABIDE—showed parity between pretrained (0.653) and non-pretrained (0.661) models, likely attributable to its larger sample size (N = 869), which provided sufficient training data even without pretraining. This dichotomy suggests that TR pretraining’s benefits are most pronounced when training data is limited, while its marginal utility diminishes with larger datasets. Importantly, AUC’s robustness to class imbalance makes these findings particularly reliable for clinical applications where class distributions are often skewed. On average, pretrained models outperformed non-pretrained counterparts by 3.8% AUC across all datasets (excluding ABIDE), reinforcing TR pretraining as a valuable strategy for neuroimaging classification tasks, though its efficacy is context-dependent.

By comparing the mean coefficient values of latent features across classes for pretrained and non-pretrained models, we assess the effectiveness of TR in improving model interpretability and performance in clinical neuroimaging applications.

To further validate the effectiveness of the learned latent representations, we conducted statistical tests on the latent features extracted before probing. Specifically, we performed one-sample and two-sample *t*-tests to assess whether the features were statistically significant in distinguishing between the classes. The results revealed a compelling trend: models with TR pretraining produced a significantly higher number of statistically significant features in both *t*-tests compared to models trained without pretraining. This pattern was consistent across all five datasets. These findings suggest that TR pretraining enables the model to learn more meaningful and discriminative features by leveraging temporal structure in the fMRI data. As a result, the pretrained models not only perform better in classification tasks but also provide feature representations that are statistically more relevant to the underlying neurobiological differences between classes.

The interpretation of weighted mean coefficient values obtained from our probing framework provides important insights into the model’s reliance on specific brain networks for the three psychiatric conditions under study.

For Sch, in the pretrained model—fine-tuned on downstream classification tasks after being initialized with weights from TR self-supervised pretraining—we observed a consistent and biologically meaningful pattern across two independent datasets, FBIRN and BSNIP. Both datasets exhibited strong negative coefficients for the Auditory network, indicating reduced or disrupted auditory activity in Sch patients. This aligns well with prior studies linking auditory processing deficits, such as hallucinations, to the disorder. In contrast, the Default Mode Network (DMN) Sensorimotor and Cerebellar networks showed positive coefficients, suggesting relatively preserved or compensatory functional activity in HCs.

The Cognitive Control network displayed a slight inconsistency, with FBIRN showing negative coefficients and BSNIP showing a small positive value. However, when examining the broader pattern of network importance across the two datasets, the Pearson correlation coefficient was 0.9003, indicating a high degree of alignment in how the model evaluates feature importance. This strong correlation supports the conclusion that the pretrained model learns generalizable and stable neurobiological signatures of Sch, irrespective of dataset-specific differences. It is particularly noteworthy given that FBIRN and BSNIP differ in scanning protocols—FBIRN being collected under eyes-closed conditions, and BSNIP under eyes-open conditions. This contrast is evident in the Visual network, where FBIRN exhibits negative coefficients and BSNIP shows positive ones, plausibly reflecting differences in Visual network engagement due to resting-state conditions. Such variation, while expected, further reinforces the importance of accounting for acquisition protocols in cross-dataset interpretations.

In sharp contrast, when models were trained from scratch without TR pretraining, the probing results revealed substantial variability and lack of consistency across datasets. As shown and discussed in the results section, network importance patterns became unstable and often contradictory. For example, several networks, such as the Sub-cortical, Cognitive Control, and Visual networks, switched signs between FBIRN and BSNIP. Additionally, the Pearson correlation between coefficient vectors dropped to −0.67, indicating a negative correlation in network importance across datasets. This suggests that the non-pretrained models not only fail to identify consistent biomarkers but may even learn dataset-specific or spurious patterns. Furthermore, the error bars for several networks—especially Cerebellar, Auditory and Visual—were significantly larger, reflecting high variance across folds and random seeds.

Similarly, in the case of Alzheimer’s disease, the pretrained model’s probing analysis revealed biologically meaningful patterns in both the DMN and the Cerebellar network, consistent with established Alzheimer’s disease (AD) pathology. The DMN showed negative coefficients, indicating its strong association with AD patients. This finding is consistent with extensive literature demonstrating DMN disruption as a hallmark of AD, characterized by reduced functional connectivity and amyloid deposition in key regions like the posterior cingulate cortex and precuneus. The model’s ability to detect this pattern underscores its validity in capturing disease-specific neural signatures.

Moreover, the cerebellar network exhibited negative coefficients, linking it to AD. While cerebellar involvement in AD has historically received less attention than cortical changes, emerging evidence highlights its role in disease progression. Studies report cerebellar metabolic deficits, atrophy, and disrupted connectivity with cortical regions, particularly in later stages of AD. The pretrained model’s identification of cerebellar dysfunction suggests it captures broader network abnormalities beyond traditional cortical foci. By contrast, the non-pretrained model’s high variability and low correlation render its findings unreliable, as discussed before.

Extending this analysis to autism, the pretrained model’s probing results reveal that the Sensorimotor network shows negative coefficients, indicating a strong association with autism. This finding suggests that atypical sensorimotor function may serve as a key neural signature of the disorder, consistent with clinical observations of sensory processing differences and motor coordination challenges in autistic individuals. The negative weighting implies that the model identifies specific patterns of sensorimotor disruption—such as reduced connectivity or aberrant activation—as characteristic features of autism.

Notably, other major networks (e.g., default mode, cerebellar) did not exhibit similarly strong negative coefficients in this analysis. This specificity highlights the sensorimotor network’s discriminative power in the pretrained model, potentially reflecting its reliability as a biomarker for autism. The result aligns with growing evidence that sensorimotor differences are a core—though historically understudied—component of autism’s neurobiology.

These model interpretations are grounded in the physiological processes captured by the fMRI BOLD signal. The negative coefficients identified in specific networks—such as the Auditory network in schizophrenia, the DMN and Cerebellar networks in Alzheimer’s disease, and the Sensorimotor network in autism—likely reflect disorder-specific alterations in neurovascular coupling and functional hyperconnectivity or hypoconnectivity. For instance, the observed reduction in auditory network activity in schizophrenia may correspond to well-documented deficits in auditory processing and glutamatergic dysfunction within temporal regions, affecting the local hemodynamic response. Similarly, the DMN disruption in Alzheimer’s disease aligns with the amyloid deposition and reduced metabolic activity that dampen the BOLD signal in posterior cingulate and prefrontal hubs. The model’s ability to consistently capture these effects across datasets suggests that the TR-pretrained features are sensitive to pathophysiological changes in neurovascular dynamics, rather than merely learning dataset-specific noise. This underscores the value of using interpretable deep learning to map model-based findings back to established neurobiological mechanisms.

While our probing analysis revealed patterns consistent with established neurobiology, we acknowledge the limitations raised by the reviewer regarding quantitative validation. The current study relies on qualitative alignment with the literature to establish biological plausibility. A crucial direction for future work is to formally quantify these associations by correlating model-derived network importance scores with independent biomarkers. For instance, in Alzheimer’s disease, the DMN coefficient values could be correlated with amyloid-PET burden or cerebrospinal fluid τ/A$\beta_{42}$ ratios within the same cohort. Similarly, for autism, sensorimotor network coefficients could be validated against behavioral measures of sensory processing. Such analyses would robustly bridge the gap between model interpretations and clinical pathophysiology.

Furthermore, while we attribute the instability of non-pretrained models to their propensity to learn dataset-specific noise, future studies could directly quantify this variability by calculating intra-class correlation (ICC) coefficients of network activity patterns across different datasets and acquisition sites. A low ICC for non-pretrained models would provide statistical evidence for their inconsistency, while a high ICC for TR-pretrained models would further solidify their generalizability.

Together, these findings demonstrate that self-supervised pretraining using Time Reversal plays a critical role in producing models that are both predictive and interpretable. TR pretraining enables the encoding of stable latent representations that generalize across cohorts and acquisition protocols. It enables downstream classifiers to rely on biologically plausible and reproducible features, thereby enhancing trust in model predictions. Our probing framework capitalizes on this by linking latent features back to functional brain networks, offering an interpretable bridge between deep learning models and domain knowledge in neuroimaging. This contributes toward the broader goal of developing transparent and clinically applicable AI models in psychiatry.

## 5. Conclusions

This study demonstrates that incorporating temporal structure through Time Reversal self-supervised pretraining significantly enhances both the predictive performance and interpretability of deep learning models applied to fMRI-based neuropsychiatric classification. Across five datasets and three clinical conditions—schizophrenia, Alzheimer’s disease, and autism—the pretrained models not only yielded higher AUC scores but also produced more statistically significant and biologically meaningful latent features. Probing analyses revealed condition-specific disruptions in functional brain networks, consistent with known pathophysiology in the literature. Importantly, TR pretraining led to greater consistency across independent datasets, emphasizing its potential to generalize well across cohorts and acquisition protocols. By bridging the gap between black-box deep learning models and clinically interpretable biomarkers, our work highlights the promise of self-supervised learning in advancing transparent, trustworthy neuroimaging-based diagnostics.

## Figures and Tables

**Table 1:** Demographic and clinical information for the datasets used in this study. Age is presented as Mean ± Standard Deviation. The Female/Male (F/M) ratio is calculated as #Females/#Males. HCP was used only for pre-training. Abbreviations: F/M, Female-to-Male ratio.

Dataset	Healthy Controls			Patients			Medication	Diagnosis (DSM-IV)	Selection Criteria
	#	Age	F/M	#	Age	F/M			
HCP	823	29 ± 3.5	1.2	–	–	–	–	–	–
FBIRN	151	38 ± 11	0.40	160	39 ± 11	0.31	Antipsychotic	Schizophrenia, Schizoaffective	Structured clinical interview for DSM-IV-TR axis I disorders (modules A–E excluding anxiety disorders).
BSNIP	338	34 ± 12	1.5	251	38 ± 12	0.68	Antipsychotic	Schizophrenia	Structured clinical interview for DSM-IV-TR axis I disorders. (Dropped bipolar, schizoaffective, and relatives)
ADNI	433	73 ± 8	1.59	66	76 ± 7	0.65	cholinesteras inhibitors or memantine inhibitors	Alzheimer’s Disease	CN and AD groups only (excluded MCI); combined ADNI-1/2/GO/3. Excluded repeated scans;
OASIS-3	651	70 ± 9	1.4	172	76 ± 8	1.0	N/A	Alzheimer’s Disease	Selection: Cognitively Normal (no AD family history) vs. AD patients. Exclusion: Repeated scans; kept earliest scan per subject.
ABIDE-I	398	17 ± 8	0.21	471	17 ± 8	0.13	N/A	Autism, Asperger’s, PDD-NOS	ABIDE I was aggregated over multiple sites, with some simple outlier filtering (e.g., based on FIQ score deviation).

**Table 2:** Statistical significance of latent features across datasets: The table demonstrates that pretrained models consistently extract more statistically significant latent features than non-pretrained counterparts across all datasets. For FBIRN, pretraining increased significant features from 156 to 196 (25.6% improvement) in one-sample tests and from 141 to 188 (33.3% improvement) in two-sample tests, revealing enhanced signal detection and group discrimination capabilities. This pattern holds across datasets, with particularly strong gains in ADNI (82 more discriminative features) and BSNIP (51 more), while ABIDE shows more modest improvements.

Dataset	Model Type	Total Features	One-Sample *t*-Test	Two-Sample *t*-Test
FBIRN	Pretrained	200	196	188
FBIRN	Non-Pretrained	200	156	141
BSNIP	Pretrained	200	195	176
BSNIP	Non-Pretrained	200	179	125
ADNI	Pretrained	200	189	182
ADNI	Non-Pretrained	200	164	100
OASIS	Pretrained	200	190	193
OASIS	Non-Pretrained	200	177	144
ABIDE	Pretrained	200	198	185
ABIDE	Non-Pretrained	200	179	131

## Data Availability

The data utilized in this study were sourced from various public repositories and in-house datasets. These included one open-source dataset: the HCP 1200 release https://www.humanconnectome.org/study/hcp-young-adult/data-releases, accessed on 7 December 2020. Additionally, two in-house datasets were used: FBIRN https://pmc.ncbi.nlm.nih.gov/articles/PMC4651841/, accessed on 7 December 2020 and B-SNIP1 https://nda.nih.gov/edit_collection.html?id=2274, accessed on 7 December 2020. The remaining three datasets (ABIDE, ADNI, and OASIS) are properly referenced in the body of the paper. All datasets underwent the NeuroMark preprocessing framework at the Center for Translational Research in Neuroimaging and Data Science (TReNDS). Due to patient data privacy regulations, the above-mentioned datasets cannot be shared publicly from TReNDS. Interested readers are encouraged to contact the respective repositories through a formal application process to request access. The NeuroMark codes have been integrated into the GIFT https://trendscenter.org/software/gift/, accessed on 7 December 2020, which is freely available for download and use by researchers worldwide. The code used for experiments presented in this study is available in our GitHub repository at NeuroLatentSignatures https://github.com/zafariqballevi2/NeuroLatentSignatures.git, accessed on 7 December 2020.

## Abbreviations

The following abbreviations are used in this manuscript:

TR	Time reversal
ICA	Independent component analysis
XAI	Explainable AI
AD	Alzheimer’s disease
ASD	Autism spectrum disorder
DMN	Default mode network
Sch	Schizophrenia
PTR	Pretrained (with Time Reversal)
NPT	Non-pretrained
